# Transcriptome modulation by hydrocortisone in severe burn shock: ancillary analysis of a prospective randomized trial

**DOI:** 10.1186/s13054-017-1743-9

**Published:** 2017-06-16

**Authors:** Jonathan Plassais, Fabienne Venet, Marie-Angélique Cazalis, Diane Le Quang, Alexandre Pachot, Guillaume Monneret, Sylvie Tissot, Julien Textoris

**Affiliations:** 1 EA7426, Université Claude Bernard Lyon 1, Hospices Civils de Lyon, bioMérieux ; “Pathophysiology of injury induced immunosuppression (PI3)”, hôpital E. Herriot, 5 place d’Arsonval, 69437 Lyon, France; 2 Hospices Civils de Lyon, Immunology laboratory, hôpital E. Herriot, 5 place d’Arsonval, 69437 Lyon, France; 3 Hospices Civils de Lyon, Burn ICU, Anesthesia and Critical Care Medicine department, hôpital E. Herriot, 5 place d’Arsonval, 69437 Lyon, France

**Keywords:** Shock, Burns, Hydrocortisone, Transcriptome modulation, Immunosuppression, Host response

## Abstract

**Background:**

Despite shortening vasopressor use in shock, hydrocortisone administration remains controversial, with potential harm to the immune system. Few studies have assessed the impact of hydrocortisone on the transcriptional response in shock, and we are lacking data on burn shock. Our objective was to assess the hydrocortisone-induced transcriptional modulation in severe burn shock, particularly modulation of the immune response.

**Methods:**

We collected whole blood samples during a randomized controlled trial assessing the efficacy of hydrocortisone administration in burn shock. Using whole genome microarrays, we first compared burn patients (n = 32) from the placebo group to healthy volunteers to describe the transcriptional modulation induced by burn shock over the first week. Then we compared burn patients randomized for either hydrocortisone administration or placebo, to assess hydrocortisone-induced modulation.

**Results:**

Study groups were similar in terms of severity and major outcomes, but shock duration was significantly reduced in the hydrocortisone group. Many genes (n = 1687) were differentially expressed between burn patients and healthy volunteers, with 85% of them exhibiting a profound and persistent modulation over seven days. Interestingly, we showed that hydrocortisone enhanced the shock-associated repression of adaptive, but also innate immunity.

**Conclusions:**

We found that the initial host response to burn shock encompasses wide and persistent modulation of gene expression, with profound modulation of pathways associated with metabolism and immunity. Importantly, hydrocortisone administration may worsen the immunosuppression associated with severe injury. These data should be taken into account in the risk ratio of hydrocortisone administration in patients with inflammatory shock.

**Trial registration:**

ClinicalTrials.gov, NCT00149123. Registered on 6 September 2005.

**Electronic supplementary material:**

The online version of this article (doi:10.1186/s13054-017-1743-9) contains supplementary material, which is available to authorized users.

## Background

Transplantation, inflammatory, and auto-immune diseases have benefitted from the immunomodulatory properties of glucocorticoids for decades. Glucocorticoids modulate both innate and adaptive immune responses [1]. They promote bone marrow release and survival of neutrophils. Glucocorticoids also modulate the innate response by suppressing pro-inflammatory or by stimulating anti-inflammatory mediators [2]. Such balanced action promotes resolution of inflammation and prevents overshooting of the host response. This may contribute to the efficacy of dexamethasone in preventing morbidity and mortality in pneumococcal meningitis in children [3]. Moreover, decreasing antigen presentation and co-stimulation by dendritic cells [4] prevents the crosstalk between innate and adaptive systems. Glucocorticoids also promote a shift from T helper (Th)1 to Th2 cells, leading to impaired defense against intracellular and opportunistic infections [5, 6].

The use of glucocorticoids in septic shock is an issue of incredible debate [7]. Considering the aforementioned effects, it seems logical that glucocorticoids may avoid the deadly effect of the massive inflammatory response initially seen in sepsis. However, after acknowledging that high doses of glucocorticoids do not decrease mortality and may even be harmful [8, 9], low (but still supra-physiologic) doses of hydrocortisone were assessed. While the reduction in mortality [10] is still a matter of debate [11, 12], hydrocortisone remains recommended for patients with refractory shock [13, 14]. Indeed, hydrocortisone has almost always been associated with reversal of shock. The beneficial effect of corticosteroids on hemodynamics is highly intertwined with their effects on the inflammatory response and endothelium [15]. Although the precise molecular mechanisms are still largely unknown, nitric oxide (NO) synthesis seems to play a determinant role in modulating the vascular tone over the initial course after injury [16, 17].

For glucocorticoids, as for any other therapies evaluated for use in septic shock, the high heterogeneity of patients and the absence of stratification may explain inconclusive results. Given the amount of evidence supporting sepsis-induced immunosuppression [18], the blind use of hydrocortisone - i.e. without monitoring the patient’s host response - may be questioned. Indeed, a retrospective study in pediatrics suggested that septic shock patients treated with glucocorticoids had numerous alterations of adaptive immunity at the transcriptional level [19].

Severe burns and septic shock host responses share numerous features, including a relative late-stage state of immunosuppression. We recently described in a prospective, randomized, double-blind study that the use of hydrocortisone in refractory burn shock led to a statistically significant reduction in the duration of shock [20]. As the impact of low-dose hydrocortisone on the host response has never been studied in patients with burns, we took advantage of this study to assess the whole blood transcriptional modulation in severe burn shock. Here, we studied the modulation of the immune response induced by shock and assessed the specific effects of hydrocortisone administration. We provide evidence that hydrocortisone transcriptionally enhances the immunosuppressive mechanisms that take place after severe injury.

## Methods

### Patients and sample collection

Patients with severe burns were admitted to Edouard Herriot Hospital (Lyon, France) and included in a placebo-controlled, randomized, double-blind study that has been described elsewhere [20]. Patients aged between 18 and 75 years, with a total burn surface area >30% were included if they presented with onset of severe shock (norepinephrine >0.5 μg/kg/min) between 24 and 72 h after injury. Pregnancy, trauma, initial sepsis and cardiac insufficiency were exclusion criteria. The protocol was accepted by the ethical committee on 15 February 2005 and was registered at ClinicalTrials.gov (NCT00149123). All healthy volunteers (HV) and patients (or next of kin) gave written informed consent before inclusion in the study.

Thirty-two patients were enrolled in the clinical study. Patients received a priming dose of 50 mg of hydrocortisone (Upjohn, Serb Labo, Paris, France) or placebo (NaCl 0.9%) followed by a continuous infusion of 200 mg/day over 5 days, 100 mg at day 6 and 50 mg at day 7, as initially proposed for septic shock [10]. Thirteen HV were recruited within Hospices Civils de Lyon to serve as controls for the transcriptional study.

Whole blood samples were collected in PAXgene Blood RNA tubes (PreAnalytix, Hilden, Germany) during the randomized controlled trial [20]. Up to four samples were collected from each patient during the first week after burn shock. The first sample (S) was collected at inclusion (onset of shock) and before any treatment (day 0, S1). Three other samples were collected the following day (24 h, S2), around 120 h (S3) and 168 h (S4) after inclusion (see details in Additional file 1: Figure S1A). Due to technical reasons (missing sample, poor RNA quality, microarray removed from quality-check analysis for batch effect, etc.), 117 samples were analyzed: 30 S1 samples, 27 S2 samples, 29 S3 samples, 18 S4 samples, and 13 samples from HV.

### RNA extraction and microarrays

Total RNA was extracted using the PAXgene™ Blood RNA kit (PreAnalytix, Hilden, Germany). Whole blood from PAXGene™ tubes was preferred to either buffy coat or peripheral blood mononuclear cells (PBMCs) to ensure reproducibility and avoid missing samples within the context of a clinical study. RNA integrity and quality were assessed using the Agilent 2100 Bioanalyser (Agilent Technologies, Waldbrom, Germany) and Lab-on-chip RNA 6000 Nano Assay (Agilent Technologies). Double-stranded complementary DNA (cDNA) was prepared from total RNA and an oligo-dT primer using GeneChip One-Cycle cDNA Synthesis Kit (Affymetrix, Santa Clara, USA). Labeled cRNA (3 μg) were hybridized onto Human Genome U133 Plus 2.0 GeneChips (Affymetrix), revealed and washed using FS450 fluidic station. GeneChips were scanned using a 5G scanner (Affymetrix) and images (DAT files) were converted to CEL files using GCOS software (Affymetrix).

### Microarray analysis

Microarray normalization and statistical analysis were performed using R/Bioconductor (R v3.0.0) [21, 22]. Quality assessment was performed using the *simpleaffy* (v2.36.1) [23] and *arrayQualityMetrics* (v3.14.0) [24] packages. The *simpleaffy* package provided quality controls before the normalization process by checking density distributions, estimating the average background intensities and assessing the RNA quality measures. The *arrayQualityMetrics* package generated a report of quality metrics from both raw data and normalized microarray data. We used principal component analysis [25] to identify poor-quality arrays. After removing outlier samples the raw data were normalized, adjusted for background noise and summarized using the guanine cytosine robust multi-array (GCRMA) algorithm with default parameters [26]. Batch effects were removed using COMBAT [27]. Filtering was performed based on MAS5.0 *p* values. MAS5.0 *p* values were estimated for each probe set, corresponding to significant differences between perfect match and mismatch intensities. Then we kept only probe sets for which all *p* values were <0.2. We finally applied a batch correction using COMBAT to remove the effect of varying delays between admission and each sampling. MIAME-compliant microarray data are available on the Gene Expression Omnibus (GEO) website [GEO:GSE77791].

### Microarray statistical analyses

The first analysis compared samples obtained from patients before they received any treatment to samples from HV. For all probe sets we performed moderated *t* tests (*Limma* package version 3.16.0) [28]; we adjusted the *p* values for multiple testing using the Benjamini-Hochberg correction [29] to ensure a false discovery rate below 5%. Adjusted *p* values <0.05 were considered significant. We then assessed gene modulation associated with burn shock across time, using only microarrays from patients on placebo. We performed moderated *F* tests [28] and corrected for multiple testing using the Bonferroni correction. Adjusted *p* values <0.05 were considered significant. Finally we assessed the impact of hydrocortisone on gene expression over time. We first performed moderated *t* tests to compare the two groups at each sampling (Benjamini-Hochberg correction [29], adjusted *p* value <0.05) and then moderated *F* tests comparing microarrays over time (S2–S4) from hydrocortisone and placebo patients. A *k*-means clustering was performed to group probe sets with similar profiles and heatmaps were used to visualize the differential expression.

### Functional annotation analyses

Functional analyses were performed using Ingenuity Pathway Analysis (IPA) software (Ingenuity Systems, Redwood City, USA) and Gene Ontology (GO) [30]. For the core analysis all differentially expressed probe set ID were mapped to their corresponding gene and molecular objects in the IPA Knowledge Base. Fisher’s exact test was used to estimate *p* values for diseases and biological function enrichment analyses, and the *p* values were adjusted using the Benjamini-Hochberg method [29]. To assess statistical significance in pathway analyses, we used repeated measure analysis of variance (ANOVA), with one between-group variable (treatment) and two within-group variables (time and probe) with the following model:

$$ \mathrm{Expression}\_\mathrm{value} \sim \mathrm{Treatment} + \mathrm{Time} + \mathrm{Probe} + \mathrm{Treatment}:\mathrm{Time} $$

The modulation of pathway expression over time and according to treatment was considered significant for *p* values <0.01.

## Results

### Patients

Briefly, 32 patients with severe burn shock were randomized to receive either hydrocortisone or placebo within the 72 h after admission. All samples from two patients were discarded due to a major batch effect related to a technical issue during hybridization. A full clinical description of the patients is provided elsewhere [20]. Briefly (Table 1), these were severely burned patients (median total burned surface area of 70% (48–84%)). Five patients (one from the placebo group and four from the hydrocortisone group) died during the first 7 days. Fewer patients in the hydrocortisone group received etomidate before ICU admission (n = 5 patients (36%) vs. n = 12 patients (80%); *p* value = 0.03). Importantly, hydrocortisone-treated patients had a significantly shorter duration of shock, defined by norepinephrine administration time (median (IQR), 60 h (42–117 h) vs. 120 h (84–141 h); *p* value = 0.048).

**Table 1:** Clinical description of the study cohort

Variable	Hydrocortisone (n = 15)	Placebo (n = 15)	Total (n = 30)	*P* value
Age, years	47 (42–50)	48 (36–59)	48 (39–55)	0.72
Gender, number of female patients (%)	2 (13%)	6 (40%)	8 (27%)	0.22
Weight (usual), kg	73 (68–86)	80 (65–93)	78 (65–86)	0.84
Weight at inclusion, kg	94 (77–98)	100 (77–112)	94 (77–104)	0.25
TBSA (%)	75 (54–87)	70 (44–76)	70 (48–84)	0.18
Baux score	117 (103–129)	109 (102–119)	110 (102–125)	0.29
ABSI score	12 (11–13)	11 (10–12)	11 (10–12)	0.06
Inhalation injury, *n* (%)	9 (60%)	3 (20%)	12 (40%)	0.06
Interval (burn injury-inclusion), h	57 (52–66)	46 (41–58)	54 (42–62)	0.11
Etomidate injection prior to inclusion, *n* (%)	5 (36%)	12 (80%)	17 (58%)	0.03
Blood transfusions before inclusion, *n* (%)	4 (27%)	3 (20%)	7 (23%)	1
Diuresis prior to inclusion (L/day)	3.2 (2.0–4.2)	3.5 (2.8–4.3)	3.4 (2.2–4.3)	0.47
Plasma creatinine prior to inclusion, μmol/L	86 (78–128)	88 (59–100)	87 (72–105)	0.28
Plasma protein prior to inclusion, g/L	42 (38–45)	41 (39–45)	42 (38–46)	1
Hemoglobin prior to inclusion, g/L	106 (100–117)	113 (90–132)	110 (94–123)	0.72
White blood cells prior to inclusion, 10^9^/L	5.0 (3.2–7.4)	6.6 (3.5–11.5)	5.3 (3.1–9.6)	0.25
Lymphocytes prior to inclusion, 10^9^/L	0.9 (0.6–1.1)	1.1 (0.8–1.4)	0.9 (0.7–1.3)	0.14
Basal cortisol, µg/dL	15.2 (8.8–21.8)	13.8 (8.4–19.1)	14.5 (8.3–19.9)	0.77
Norepinephrine prior to inclusion, μg/kg/min	0.59 (0.53–0.66)	0.60 (0.51–1.04)	0.60 (0.51–0.78)	0.55
Duration of norepinephrine protocol, h	60 (42–117)	120 (84–141)	102 (56–131)	0.05
Total quantity of norepinephrine over ICU stay, μg/kg	1457 (1132–3705)	1971 (1535–3893)	1771 (1196–4068)	0.27
Septic shock, *n* (%)	5 (33%)	7 (47%)	12 (40%)	0.71
Number of infections	1 (1–3)	2 (2–3)	2 (1–3)	0.1
Number of skin grafts	4 (1–7)	5 (4–10)	5.00 (1.3–8.5)	0.13
Duration of hospitalization, days	65 (11–77)	67 (51–104)	66 (22–89)	0.11
Deaths before 28 days, *n* (%)	6 (40%)	2 (13%)	8 (27%)	0.22

*TBSA* total burn surface area, *ABSI* Abbreviated Burn Severity Index

### Host response signature to burn shock over time

To study the host response to burn shock over time, inclusion samples (S1) were compared to 13 HV samples. Then, to study the modulation over the first week, we analyzed only samples from the placebo group (the complete analysis plan is provided in Additional file 1: Figure S1B).

**Fig. 1:**
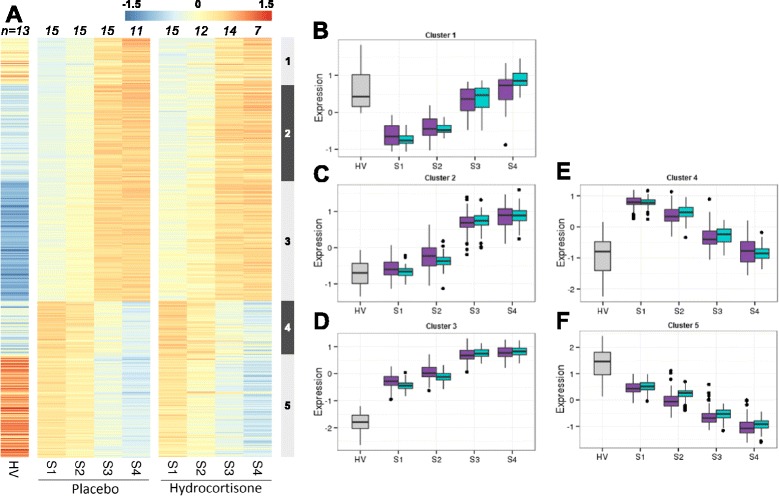
Modulation of gene expression after burn shock. **a** Heatmap representation of expression in genes modulated by burn injury over time. Gene expression (*rows*) is color-coded from *blue* to *orange*. Individual values were averaged according to sample time (*columns*), and the associated number of samples is presented above each column. Although this analysis was performed only in patients on placebo, we included the representation of the identified gene modulation in hydrocortisone-treated patients alongside. Five (1 to 5) clusters were identified by unsupervised analysis, according to a similar longitudinal profile of expression, and are illustrated as boxplots (**b**-**f**). Hydrocortisone-treated patients are in *purple* and placebo-treated patients are in *green. S1* to *S4* sample times day 0, day 1, day 5 and day 7, respectively, *HV* healthy volunteers

At inclusion, 1510 probe sets (967 genes) were differentially expressed between patients and HV (23.5% of the tested set). We observed two distinct temporal patterns of gene modulation: 464 probe sets (266 genes, 15%, clusters 1, 4) were transiently modulated whereas 2107 probe sets (1687 genes, 85%, clusters 2, 3 and 5) were persistently modulated (Fig. 1 and Additional file 2: Table S2). Genes from clusters 1 and 4 were initially down-modulated and up-modulated, respectively, and returned to HV levels within the first week. Functional annotation of these transiently modulated genes highlighted interesting functions (Additional file 3: Table S1). Genes involved in the response to hypoxia were down-modulated, whereas several processes related to inflammation and blood vessels were up-modulated.

Modulation of genes from cluster 3 (up-modulated), and 5 (down-modulated) increased over time and was maximal at the last time point. Genes from cluster 2 were up-modulated only at later time points (days 5 and 7 after inclusion). Functional annotation of cluster 5 (down-modulated) highlighted functions related to T cell differentiation and activation, negative regulation of apoptotic process, regulation of the innate immunity and antigen presentation. Interestingly, annotation of the persistently up-modulated genes (clusters 2 and 3) highlighted several genes involved in response to stress, mitochondrial respiratory chain and oxidative phosphorylation (Additional file 3: Table S1). Altogether, we observed a profound and persistent modulation of gene expression after severe burn shock.

### Modulation of gene expression by hydrocortisone after burn shock

**Fig. 2:**
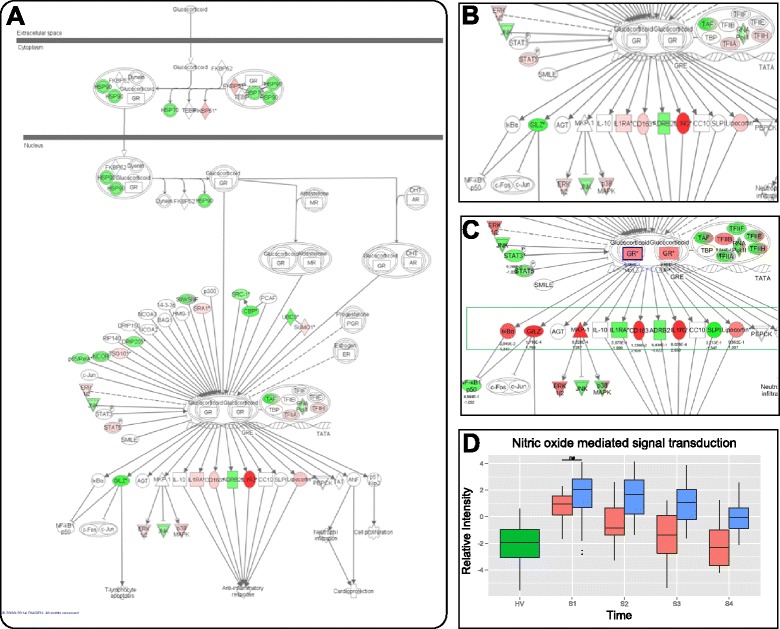
Modulation of the glucocorticoid receptor and nitric oxide signaling pathways. **a** Graphical representation of the glucocorticoid receptor (*GR*) pathway was performed through Ingenuity Pathway Analysis. The relative modulation of gene expression between burn patients and healthy volunteers (*HV*) is color coded from *green* (the gene is less expressed in patients with burns) to *red* (the gene is more expressed in patients with burns). **b**, **c** To focus on the effect of hydrocortisone administration, a zoom was performed on the targets of the glucocorticoid receptor, according to analysis 1 (placebo-treated burn patients vs. HV (**b**)) or analysis 2 (hydrocortisone-treated patients vs. placebo-treated patients (**c**)). **d** Summary of the effect of hydrocortisone administration on the gene expression for the nitric oxide mediated signal transduction pathway [GO:0007263] in HV (*green*), hydrocortisone-treated patients (*red*) or placebo-treated patients (*blue*). Statistical significance of the observed differences was assessed using repeated measures analysis of variance taking into account treatment, time and gene (probes) effects. We observed significant modulation over time (*p* value *< 10* ^*-6*^), and according to hydrocortisone administration (*p* value *< 10* ^*-6*^). *S1* to *S4* sample times day 0, day 1, day 5 and day 7, respectively

At inclusion, burn patients exhibited moderate to high levels of plasma cortisol, without a difference between hydrocortisone and placebo groups (respectively, 15 μg/dL (8.8–22) and 14 μg/dL (8.4–19); *p* value = 0.77). The expression of the glucocorticoid receptor (GR) was similar in patients and healthy volunteers (Fig. 2a; *NR3C1* (alias GR): FC = 0.88, *p* value = 0.42). Most patients exhibited relative adrenal insufficiency, with small increases in plasma cortisol levels after adrenocorticotropic hormone (ACTH) stimulation (Table 1). This was consistent with global down-modulation of the GR pathway genes at inclusion (Fig. 2a and b).

**Fig. 3:**
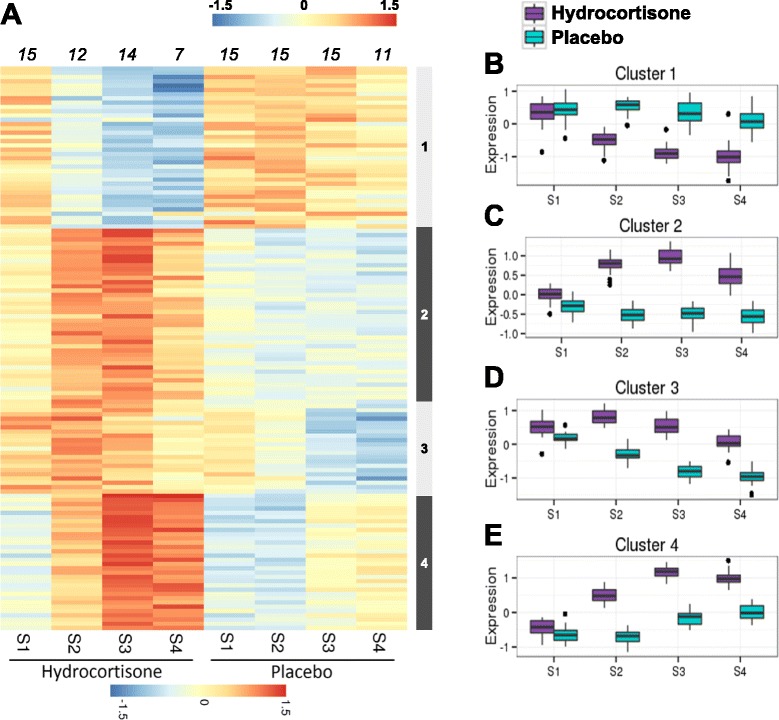
Modulation of gene expression induced by hydrocortisone administration after burn shock. **a** Heatmap representation of expression in genes specifically modulated by hydrocortisone. Gene expression (*rows*) is color-coded from *blue* to *orange*. Individual values were averaged according to sampling time (*columns*), and the associated number of patients is presented above each column. Four (1 to 4) clusters were identified by unsupervised analysis, according to a similar longitudinal profile of expression, and illustrated as boxplots (**b**-**e**). Hydrocortisone-treated patients are in *purple* while placebo-treated patients are in *green. S1* to *S4* sample times day 0, day 1, day 5 and day 7, respectively, *HV* healthy volunteers

Interestingly, hydrocortisone induced only a transient modulation of gene expression over the seven days of treatment. Indeed, only 246 probesets (175 genes) were modulated after hydrocortisone administration (Fig. 3b-e), and 27 probesets (22 genes) were still differentially expressed between the two groups at the last time point (Additional file 2: Table S2B). Hydrocortisone up-modulated most of the genes of the GR pathway (clusters 2 to 4, Fig. 3c-e), including the GR itself, and several of its targets (Fig. 2c).

### Hydrocortisone modulation of vascular tone at the transcriptional level

As hydrocortisone has been shown to reduce the duration of septic shock [10, 31] and severe burn shock [20], we explored selected mechanisms involved in vascular tone control: the modulation of adrenergic and NO pathways at the transcriptional level. As shown in Fig. 2a and b, *ADRB2* (β2-adrenoreceptor), a direct target of the GR, was down-modulated in patients in comparison to HV. However, its expression was further down-modulated after hydrocortisone treatment (Fig. 2c). We also explored alpha-adrenergic receptors, and found no difference between hydrocortisone-treated and placebo-treated groups. Although NO synthase genes were not modulated (*NOS1, NOS2* and *NOS3*), we found that the “NO mediated signal transduction pathway” [GO:0007263] was significantly down-modulated by hydrocortisone (S2, S3, and S4, *p* value < 10^-6^ ; Fig. 2d).

### Modulation of the host response toward immunosuppression

**Fig. 4:**
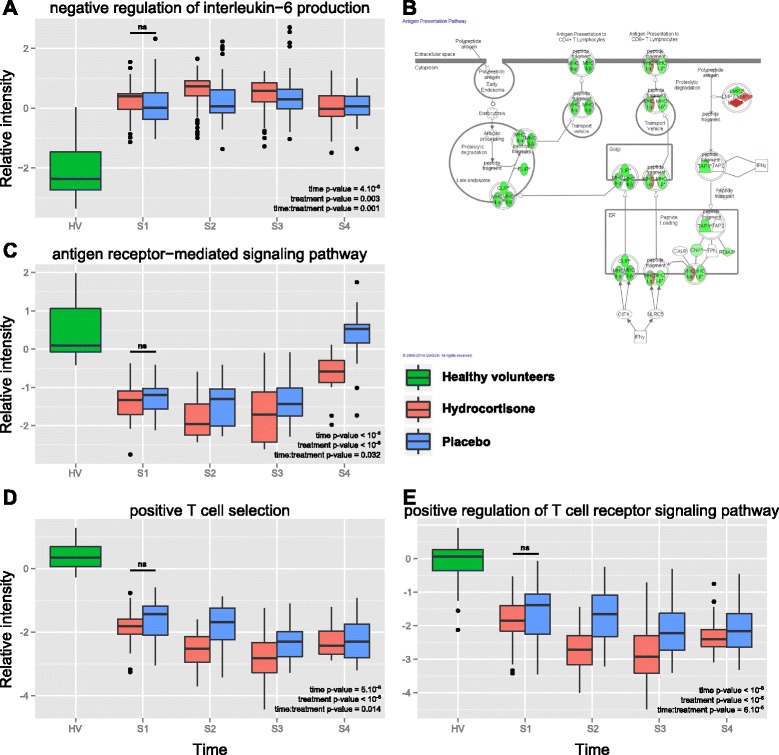
Modulation of the immune response by hydrocortisone. Summary of the effect of hydrocortisone administration on gene expression in healthy volunteers (*green*), hydrocortisone-treated patients (*red*) or placebo-treated patients (*blue*) for various components of the immune response. **a**. Negative regulation of IL-6 production [GO:0032715]. **c** Antigen receptor-mediated signaling pathway [GO:0050851]. **d** Positive T cell selection [GO:0043368]. **e** Positive regulation of the T cell receptor signaling pathway [GO:0050862]. The statistical significance of the observed differences was assessed using repeated measures analysis of variance, taking into account treatment, time and gene (probes) effects. **b** Ingenuity Pathway Analysis® representation of the antigen presentation pathway, where components are color coded according to relative gene expression between hydrocortisone-treated patients and placebo-treated patients at S4. *S1* to *S4* sample times day 0, day 1, day 5 and day 7, respectively, *HV* healthy volunteers, *ns* not significant

To explore the modulation of immune functions by hydrocortisone in the context of burn shock, we extracted several immune response pathways from Gene Ontology (GO). We summarized gene expression for each process and plotted scaled-centered values according to time and hydrocortisone administration. As shown in Fig. 4a, b and c, innate response was impaired, with significant negative regulation of pro-inflammatory cytokines (e.g. IL-6, GO:0032715; *p* value = 3.10^-3^), and negative regulation of antigen-receptor-mediated pathway (GO:0050857; *p* value < 10^-6^). T cell response was also affected by hydrocortisone, as shown by significant down-modulation of genes involved in both positive T cell selection (GO:0043368, Fig. 4d; *p* value < 10^-6^), and regulation of T cell receptor pathway (GO:0050862, Fig. 4e; *p* value < 10^-6^).

## Discussion

Here, we took advantage of a prospective, randomized, double-blind study to assess whole blood transcriptional modulation in severe burn shock and according to hydrocortisone administration. We identified wide and persistent modulation of gene expression over the first week after shock, whereas hydrocortisone-associated transcriptional modulation was moderate and transient. We also characterized the impact of both shock and hydrocortisone on the immune response, and showed that hydrocortisone transcriptionally enhanced the immunosuppressive mechanisms that occur after severe burn injury.

Few studies have evaluated the transcriptional host response to burn injury in humans. Modulation of gene expression after burn in skeletal muscle [32, 33], skin [34, 35], and adipose tissue [36] leads to identification of pathways involved in metabolism, cell proliferation and inflammatory response, and osteogenic differentiation of mesenchymal cells, respectively. Regarding the blood transcriptome of burn patients, a first dataset (GSE19743, buffy-coat samples) from 57 patients and 63 healthy volunteers (analyzed in [37–39]) showed that genes modulated within 10 days after injury were mainly related to immunity. Genes modulated at a later stage (11–49 days) were also involved in metabolism and apoptosis [39]. Our results were consistent regarding the identified functional pathways. We showed that modulation was triggered early after burns as most genes were already modulated within 72 h of injury. A second dataset described the pooled-leukocytes transcriptome of 112 burn patients sampled within 7 days [40]. This study assessed prognostic factors and found a 39-gene signature associated with a burn size >40%. These genes were related to platelet activation, TNF production, cellular adhesion, migration, and degranulation. Interestingly, our dataset shared 8 out of the 10 most modulated genes (*LCN2, LTF, THBS1, ITGA2B, CD24, TCN1, BPI* and *SLC51A*). Finally, we observed that most of the differentially expressed genes exhibited sustained modulation over time after burn injury. This is similar to a previous observation by Seok et al., whereby burn patients exhibited the longest period of transcriptome “recovery time” [41].

The beneficial effect of hydrocortisone on the duration of septic shock is now widely accepted. We recently demonstrated a similar effect in burns, i.e. non-infectious/inflammatory shock [20]. Glucocorticoids play important roles in the modulation of vascular tone. Although glucocorticoid-induced hypertension is primarily due to sodium retention and volume expansion, an increase in peripheral vascular resistance may also play a role [42]. Here, we found no difference in adrenergic receptor expression in hydrocortisone-treated vs. placebo-treated patients. Several limitations might explain this negative result. First, the effects of hydrocortisone might be tissue-specific and not seen in the whole blood transcriptome. Second, the GR is known to have both genomic and non-genomic effects [43], the latter being involved in the density of adrenergic receptors in vessels [44] and the modulation of agonist-induced contractions in vascular smooth muscle at multiple sites along signal transduction pathways.

Glucocorticoid receptor may modulate other pathways involved in vascular tone such as NO. Indeed, we observed that the NO-mediated signal transduction pathway was up-modulated early after burn, but returned to control values quicker in hydrocortisone-treated than in placebo-treated patients. This result is consistent with former literature, as mice lacking endothelial GR were found to have higher levels of NO, and increased hemodynamic instability [15]. Moreover, hydrocortisone administration to patients with septic shock was associated with reduction in plasma nitrite/nitrate (indicative of lower NO formation) and of vasopressor support [17]. Taken together, these results are in favor of a hydrocortisone-induced modulation of NO balance that may explain positive hemodynamic effects in shock patients.

Glucocorticoids also have many side effects such as hyperglycemia, critical illness polyneuromyopathy [45], delayed wound healing, and immunosuppression. These side effects and the absence of convincing results for the effect on mortality may explain the wide heterogeneity of practice in hydrocortisone administration in septic shock. Wong et al. have recently shown in pediatric patients with septic shock that corticosteroid administration was associated with greater repression of adaptive immunity-related genes [19]. Despite well-matched groups in terms of severity, this study was retrospective, with no control over sampling time. Here, we confirmed the impact of hydrocortisone administration on host immune response in a different, but close model of inflammatory shock. Moreover, our prospective and randomized design allowed us to follow the hydrocortisone-related modulation of gene expression over a week.

Interestingly, along with greater repression of adaptive immunity, we also observed an impact of hydrocortisone on innate immunity. Indeed, the down-modulation of the antigen receptor-mediated pathway (Fig. 4b-c) was significantly greater at day 7 in the hydrocortisone group. This result was reminiscent of repressed monocyte expression of HLA-DR seen in various acute inflammatory responses, including burns, where it was associated with the occurrence of secondary septic shock [46]. These results underline that hydrocortisone administration may deepen the immunosuppression associated with severe injury.

Interestingly, other groups have reported beneficial effects of hydrocortisone administration in injury-related models. In severe trauma, the incidence of hospital-acquired pneumonia was lower in the hydrocortisone group [47]. The author’s hypothesis was that early hydrocortisone administration could blunt the hyper-inflammatory response associated with trauma, and prevent the subsequent associated immunosuppression. However, these results were not confirmed in a second multicenter trial published recently [48]. In combination with our current results, this raises the question of: (1) the timing of hydrocortisone administration after injury, and (2) the duration of hydrocortisone administration. This also underlines the lack of tools to identify/stratify patients who may benefit from hydrocortisone.

Our study has several limitations. Despite an adequate design, the small sample size precluded us from assessing associations between hydrocortisone, host-response and outcomes such as mortality or secondary infections. As we selected only patients with severe shock (with >0.5 μg/kg/min norepinephrine), most of them had extensive burns (median TBSA = 70% (48–84), Table 1) and we found no transcriptional modulation according to TBSA. Therefore, we cannot extrapolate to the host response modulation in every patient with burns. However, this provided us with a very homogeneous cohort of patients, allowing us to more precisely decipher the pathways modulated after severe burn injury, and to identify similarities with inflammatory situations such as trauma and septic shock [49]. As described in Table 1, several confounding factors might have impacted the transcriptome modulation over time (ABSI, etomidate administration, etc.). We observed no significant difference in the results when adjusting or not adjusting with these variables but the small sample size precludes a definitive conclusion. As all patients received blood transfusion during graft surgery, the impact of transfusion on transcriptome modulation could not be assessed. This deserves more specific evaluation in the future. Moreover, as we did not collect whole blood cell counts except at admission, we were not able to verify if changes in the pattern of blood leukocytes may have impacted longitudinal gene expression. Surprisingly, hydrocortisone treatment was only associated with a few modulated genes. Our small sample size and stringent thresholds for probe set filtering might explain such results. An additional explanation could be related to the profound basal modulation induced by burn injury, which might limit our ability to detect all hydrocortisone-modulated genes. However, such a design also allowed us to describe the impact of hydrocortisone on gene expression in vivo in an acute inflammatory situation, for the first time. Finally, our data were limited to mRNA expression. We were not able to test correlation with either translational modulation, or functionality of the immune system. Demonstration of altered immune functionality in burn patients is thus still pending.

## Conclusions

In conclusion, we assessed the early transcriptional modulation of the host response to burn shock and to hydrocortisone administration. The initial response to burn shock encompasses wide and persistent genomic modulation, with a profound alteration of pathways associated with metabolism and immunity. We identified down-modulation of both innate and adaptive immune responses during the first week after severe burn injury. We believe that these results support the need for more precise evaluation of the benefit/risk ratio of hydrocortisone administration in critical illness, where injury-induced immunosuppression may occur.

## Additional files

Additional file 1: Figure S1. Flowchart of the study. **a** Schematic representation of the timing of sampling during the course of administration of hydrocortisone or placebo. *d* day, *S* sample. **b** Flowchart of the study describing the number of samples analyzed for each time point, and the pre-processing steps of the bioinformatics analysis. (PDF 61 kb)

Additional file 2: Table S2. Details of modulated probe sets and genes at each time point for analysis 1, comparing modulation of gene expression according to burn injury (A). and analysis 2, comparing the modulation of gene expression according to hydrocortisone treatment (B). Each cell provides the number of modulated probe sets (genes). (DOC 40 kb)

Additional file 3: Table S1. Functional annotation of genes modulated by burn shock, classified according to their modulation pattern. (DOCX 23 kb)

## Abbreviations

ACTH: Adrenocorticotropic hormone
ADRB2: β2-Adrenoreceptor gene symbol
cDNA: Complementary Desoxyribo Nucleic Acid
GEO: Gene Expression Omnibus
GO: Gene Ontology
GR: Glucocorticoid receptor
HV: Healthy volunteers
IL: Interleukin
NO: Nitric oxide
NOS: Nitric oxide synthase
RNA: Ribonucleic acid
TNF: Tumor necrosis factor
