# Supplementary figures and images for: Transcriptome modulation by hydrocortisone in severe burn shock: ancillary analysis of a prospective randomized trial
Source: Crit Care. 2017 Jun 16;21:158. doi: 10.1186/s13054-017-1743-9 (PMC5473974; doi:10.1186/s13054-017-1743-9)

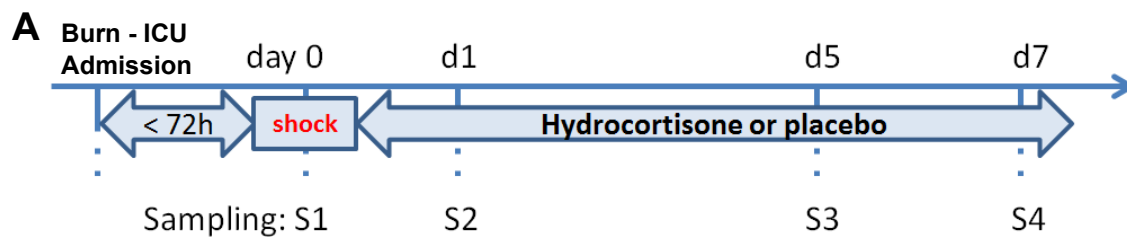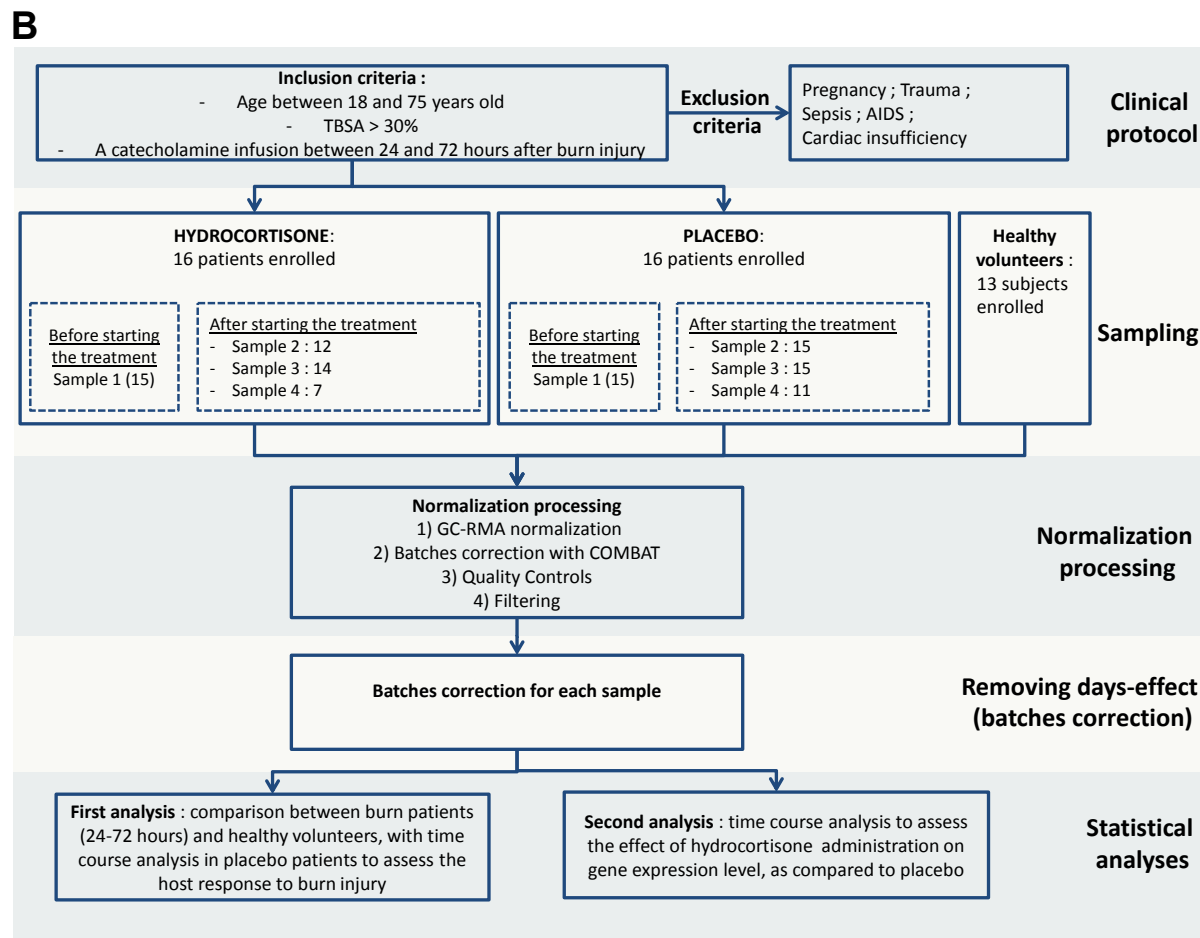

Supplement: Supplementary file 1 — Flowchart of the study. a Schematic representation of the timing of sampling during the course of administration of hydrocortisone or placebo. d day, S sample. b Flowchart of the study describing the number of samples analyzed for each time point, and the pre-processing steps of the bioinformatics analysis. (PDF 61 kb) [file 13054_2017_1743_MOESM1_ESM.pdf]
