# Supplementary material for: Transcriptome modulation by hydrocortisone in severe burn shock: ancillary analysis of a prospective randomized trial
Source: Crit Care. 2017 Jun 16;21:158. doi: 10.1186/s13054-017-1743-9 (PMC5473974; doi:10.1186/s13054-017-1743-9)
Supplement: Supplementary file 2 — Details of modulated probe sets and genes at each time point for analysis 1, comparing modulation of gene expression according to burn injury (A). and analysis 2, comparing the modulation of gene expression according to hydrocortisone treatment (B). Each cell provides the number of modulated probe sets (genes). (DOC 40 kb) [file 13054_2017_1743_MOESM2_ESM.doc]

**Supplementary Table 2:** Details of modulated probesets and genes at each time point for analysis 1 – comparing modulation of gene expression according to burn injury (A) – and analysis 2 – comparing the modulation of gene expression according to hydrocortisone treatment (B). Each cell provide the number of modulated **probesets** *(genes).*

1. Modulation of gene expression after severe burn (Burn vs Healthy volunteers ; placebo only)

|  | **T1** | **T2** | **T3** | **T4** | **TOTAL** |
| --- | --- | --- | --- | --- | --- |
| **UP modulated** | **1033** | **1094** | **1372** | **1262** | **1589** |
|  | *(645)* | *(766)* | *(778)* | *(781)* | *(1045)* |
| **DOWN modulated** | **477** | **564** | **813** | **845** | **982** |
|  | *(323)* | *(399)* | *(393)* | *(398)* | *(644)* |
| **TOTAL** | **1510** | **1658** | **2185** | **2107** | **2571** |
|  | *(967)* | *(1077)* | *(1462)* | *(1421)* | *(1687)* |

1. Modulation of gene expression according to hydrocortisone treatment

|  | **T1** | **T2** | **T3** | **T4** | **TOTAL** |
| --- | --- | --- | --- | --- | --- |
| **UP modulated** | **1** | **77** | **90** | **14** | **120** |
|  | *(1)* | *(56)* | *(63)* | *(12)* | *(87)* |
| **DOWN modulated** | **0** | **53** | **85** | **13** | **126** |
|  | *(0)* | *(33)* | *(62)* | *(10)* | *(89)* |
| **TOTAL** | **1** | **130** | **175** | **27** | **246** |
|  | *(1)* | *(89)* | *(125)* | *(22)* | *(175)* |
