# Supplementary material for: Transcriptome modulation by hydrocortisone in severe burn shock: ancillary analysis of a prospective randomized trial
Source: Crit Care. 2017 Jun 16;21:158. doi: 10.1186/s13054-017-1743-9 (PMC5473974; doi:10.1186/s13054-017-1743-9)
Supplement: Supplementary file 3 — Functional annotation of genes modulated by burn shock, classified according to their modulation pattern. (DOCX 23 kb) [file 13054_2017_1743_MOESM3_ESM.docx]

**Supplementary Table 1. Functional annotation of genes modulated by burn shock, classified according to their modulation pattern**

| **Term** | **p-value** | **Fold Enrichment** | **Benjamini p-value** |
| --- | --- | --- | --- |
| **Persistent UP** | | | |
| GO:0006091 - generation of precursor metabolites and energy | 2.22E-06 | 4.87 | 2.48E-03 |
| GO:0006119 - oxidative phosphorylation | 4.75E-05 | 8.30 | 0.03 |
| GO:0022900 - electron transport chain | 1.24E-04 | 7.14 | 0.05 |
| GO:0034654 - nucleobase, nucleoside, nucleotide and nucleic acid biosynthetic process | 6.05E-04 | 4.74 | 0.16 |
| GO:0044271 - nitrogen compound biosynthetic process | 1.31E-03 | 3.44 | 0.25 |
| GO:0042773 - ATP synthesis coupled electron transport | 2.15E-03 | 9.08 | 0.33 |
| GO:0045333 - cellular respiration | 2.58E-03 | 6.29 | 0.30 |
| GO:0033554 - cellular response to stress | 3.55E-03 | 2.52 | 0.30 |
| GO:0055114 - oxidation reduction | 3.81E-03 | 2.39 | 0.30 |
| GO:0034613 - cellular protein localization | 0.01 | 2.72 | 0.45 |
| GO:0032446 - protein modification by small protein conjugation | 0.01 | 4.62 | 0.51 |
| GO:0006259 - DNA metabolic process | 0.01 | 2.41 | 0.53 |
| GO:0048015 - phosphoinositide-mediated signaling | 0.01 | 5.78 | 0.51 |
| GO:0006260 - DNA replication | 0.01 | 3.75 | 0.49 |
| GO:0006974 - response to DNA damage stimulus | 0.01 | 2.73 | 0.47 |
| GO:0051188 - cofactor biosynthetic process | 0.01 | 5.24 | 0.48 |
| GO:0006281 - DNA repair | 0.02 | 2.87 | 0.58 |
| **Persistent DOWN** | | | |
| GO:0006955 - immune response | 1.38E-07 | 4.97 | 1.11E-04 |
| GO:0002504 - antigen processing and presentation of peptide or polysaccharide antigen via MHC class II | 5.06E-07 | 36.71 | 2.04E-04 |
| GO:0046649 - lymphocyte activation | 4.10E-04 | 7.10 | 0.08 |
| GO:0045321 - leukocyte activation | 1.14E-03 | 5.84 | 0.14 |
| GO:0045088 - regulation of innate immune response | 2.30E-03 | 14.96 | 0.19 |
| GO:0042110 - T cell activation | 3.31E-03 | 8.01 | 0.22 |
| GO:0030217 - T cell differentiation | 3.90E-03 | 12.43 | 0.22 |
| GO:0050778 - positive regulation of immune response | 0.01 | 6.96 | 0.27 |
| GO:0030097 - hemopoiesis | 0.01 | 5.13 | 0.27 |
| GO:0050870 - positive regulation of T cell activation | 0.01 | 10.63 | 0.25 |
| GO:0006952 - defense response | 0.01 | 2.95 | 0.33 |
| GO:0045449 - regulation of transcription | 0.01 | 1.71 | 0.32 |
| GO:0045582 - positive regulation of T cell differentiation | 0.01 | 18.93 | 0.32 |
| GO:0008283 - cell proliferation | 0.02 | 3.24 | 0.42 |
| GO:0045089 - positive regulation of innate immune response | 0.02 | 13.17 | 0.43 |
| GO:0016064 - immunoglobulin mediated immune response | 0.03 | 11.22 | 0.49 |
| GO:0043066 - negative regulation of apoptosis | 0.03 | 3.42 | 0.48 |
| GO:0019724 - B cell mediated immunity | 0.03 | 10.82 | 0.49 |
| GO:0045059 - positive thymic T cell selection | 0.03 | 57.69 | 0.50 |
| GO:0007242 - intracellular signaling cascade | 0.04 | 1.93 | 0.52 |
| **Transient UP** | | | |
| GO:0043433 - negative regulation of transcription factor activity | 8.17E-03 | 21.47 | 0.99 |
| GO:0042167 - heme catabolic process | 9.07E-03 | 214.73 | 0.94 |
| GO:0001525 - angiogenesis | 0.01 | 8.71 | 0.87 |
| GO:0043392 - negative regulation of DNA binding | 0.01 | 18.95 | 0.80 |
| GO:0006954 - inflammatory response | 0.02 | 4.96 | 0.72 |
| GO:0006955 - immune response | 0.02 | 3.27 | 0.68 |
| GO:0019221 - cytokine-mediated signaling pathway | 0.02 | 13.80 | 0.69 |
| GO:0007243 - protein kinase cascade | 0.03 | 4.35 | 0.75 |
| GO:0048514 - blood vessel morphogenesis | 0.03 | 6.11 | 0.73 |
| GO:0042730 - fibrinolysis | 0.04 | 53.68 | 0.82 |
| GO:0001568 - blood vessel development | 0.04 | 5.26 | 0.79 |
| GO:0051090 - regulation of transcription factor activity | 0.04 | 9.38 | 0.78 |
| GO:0001944 - vasculature development | 0.04 | 5.13 | 0.77 |
| **Transient DOWN** | | | |
| GO:0001666 - response to hypoxia | 0.04 | 9.46 | 1.00 |
| GO:0006783 - heme biosynthetic process | 0.04 | 49.74 | 1.00 |
| GO:0070482 - response to oxygen levels | 0.04 | 8.99 | 1.00 |
| GO:0006779 - porphyrin biosynthetic process | 0.04 | 42.28 | 1.00 |
